# Population pharmacokinetic analysis of transdermal granisetron in healthy Chinese and Caucasian volunteers

**DOI:** 10.3389/fphar.2023.1154026

**Published:** 2023-06-26

**Authors:** Jiayu Li, Pei Hu, Li Zhou, Fumiko Nagahama, Rui Chen

**Affiliations:** ^1^ School of Chinese Materia Medica, Beijing University of Chinese Medicine, Beijing, China; ^2^ Clinical Pharmacology Research Center, Peking Union Medical College Hospital, State Key Laboratory of Complex Severe and Rare Diseases, NMPA Key Laboratory for Clinical Research and Evaluation of Drug, Beijing Key Laboratory of Clinical PK and PD Investigation for Innovative Drugs, Chinese Academy of Medical Sciences & Peking Union Medical College, Beijing, China; ^3^ Solasia Medical Information Consulting Co, Ltd., Tokyo, Japan; ^4^ Solasia Pharma K.K., Tokyo, Japan

**Keywords:** population pharmacokinetics, granisetron, transdermal delivery system, 5-HT3 receptor antagonist, Phoenix NLME, Chinese, Caucasian

## Abstract

Granisetron patches are a prolonged delivery transdermal system that is used to prevent Chemotherapy-induced nausea and vomiting (CINV). To date, no pharmacokinetics comparison between Chinese and Caucasian populations has been conducted for granisetron patches. This study focused on the ethnic differences in pharmacokinetics (PK) of granisetron transdermal delivery system (GTDS) between Chinese and Caucasians and the influence of demographic covariates on pharmacokinetics (age, weight, height, body mass index, sex). To achieve this, blood concentration data were collected from 112 Caucasian healthy subjects participating in four clinical trials and 24 Chinese healthy subjects from one clinical trial, after a single application of the granisetron transdermal delivery system. A nonlinear mixed-effects model method of Phoenix NLME software was used to establish a population pharmacokinetic (Pop PK) model for Caucasian subjects. Bootstrap and visual predictive check (VPC) were used to validate the model. Based on the analysis a one-compartment model with first-order absorption and a first-order elimination well described the PK characteristics of GTDS. The apparent systemic clearance was determined to be 31316.3 mL/h and the central compartment volume of distribution was 6299.03 L. None of the five covariates (age, weight, height, body mass index, and sex) included in the Pop PK were significant covariates affecting PK. The final Pop PK model was used to simulate the Caucasian blood concentration by applying the dosing regimen used for the Chinese population. Comparison of the simulated Caucasian PK data with observed clinical PK data from Chinese healthy subjects revealed no significant differences in the main parameters, AUC_last_ and C_avg_, between the two groups. These findings suggested that no dose adjustment was required when applied to the Chinese population. In conclusion, this Pop PK study comparing the transdermal patch in Chinese and Caucasian healthy subjects provided valuable insights for optimizing dosage across ethnicities.

## 1 Introduction

Chemotherapy-induced nausea and vomiting (CINV) is considered one of the most distressing side effects in chemotherapy patients, which greatly impacts their quality of life and often leads to decreased adherence to medication (Coates et al, 1983). One of the main mechanisms behind CINV is the release of serotonin from enterochromaffin cells 0-24 h after chemotherapy. This serotonin activates the 5-hydroxytryptamine type 3 (5-HT3) receptor located on the vagal afferent, which transmits the stimulus to the brain (Spartinou et al, 2017). The National Comprehensive Cancer Network Clinical Practice Guidelines for Antiemetics include 5-HT3 receptor antagonists, such as Granisetron, as a preventive measure for moderate or high emetic risk treatment (Aapro, 2018).

Despite substantial advances in the prevention of CINV, studies indicate that up to 40% of cancer patients still experience symptoms after receiving chemotherapy. One of the primary reasons for ineffective CINV control is patients do not comply with guideline medications (Keating et al, 2012). The lack of adherence can be attributed to two main factors, objective dosing restrictions, and weak subjective awareness. For instance, patients with impaired oral cavities or those relying on gastric tubes for feeding are unable to take oral medication (Keating et al, 2012). Furthermore, cancer patients with psychiatric disorders have weak subjective awareness of oral medication use (Caroli et al, 2011). For the above scenarios, granisetron transdermal delivery system (GTDS) can release granisetron continuously through the skin into the circulatory system for several days after only a single dose to the forearm. This method is simple and convenient to use, allowing family members and healthcare providers to check the medication situation instantaneously. Meanwhile, GTDS bypasses the gastrointestinal route of absorption perfectly.

As a 5-HT3 receptor antagonist, granisetron was introduced to domestic and international markets in the early 1990s in oral and intravenous delivery formulations. The transdermal systemic drug “Sancuso” contains 34.3 mg/52 cm^2^ granisetron and is the first FDA-approved transdermal systemic drug for 5-HT3 receptor antagonists (Howell et al, 2009; NCCN., 2023). The patch is applied to the outer upper arm, delivering a dosage of 34.3 mg/52 cm^2^, 24-48 h before chemotherapy. It is removed at least 24 h after chemotherapy. The effectiveness of the patch can last for 7 days. After percutaneous absorption, granisetron reaches C_max_ at 48 h. The protein binding rate of GTDS is 65%, with an elimination fraction of 25%, and undergoes metabolism by liver CYP1A1 (fraction of metabolism, fm = 0.69), CYP3A4 (fm = 0.05) (Dallmann et al, 2018). The total *in vivo* exposure AUC is 420 ng/mL*h and C_avg_ is 2.6 ng/mL over 6 days. These values are comparable to the NCCN guidelines, which recommend an AUC of 306 ng/mL and C_avg_ of 2.2 ng/mL for oral administration of 2 mg granisetron over 5 days (Howell et al, 2009). A randomized, double-blind, multiple international research centers, phase III clinical trial compared the complete control rate of CINV in patch and oral dosage form. The study revealed a complete control rate of 60% for the patch and 65% for the oral formulation (Boccia et al, 2011). In terms of the complete vomiting control rate as the endpoint, the efficacy of the two medicines demonstrated similar efficacy.

Absorbed through the skin makes it special to avoid the gastrointestinal tract, but brings uncertainty when patients have various skin conditions. The factors that affect drug absorption arise from both drug itself and the subject. When the same drug is applied to different populations, the skin characteristics of subjects from different races such as skin temperature, water content of the stratum corneum, and skin thickness can influence the drug absorption process (Kompaore and Tsuruta, 1993). The current 34.3 mg/52 cm^2^ GTDS is based on clinical trials conducted in the Caucasian population (Howell et al, 2009). Ethnic differences between the Chinese and Caucasian populations at the physiological level may lead to different PK to drug. Firstly, variations in skin characteristics can lead to differences in exposure and affect drug efficacy. Research has shown that East Asians tend to have a thinner skin barrier compared to African Americans (Muizzuddin et al, 2010), the density of secretory glands in the skin is higher (Rawlings, 2006) and the skin is more sensitive to exogenous chemicals. Therefore, particular attention should be given to the occurrence of allergic events when GTDS is applied to the East Asian population. Secondly, physiological differences in body shape and elimination organs, such as the liver, may lead to differences in drug disposal, raising potential safety concerns. The body surface area (BSA) of Caucasian male volunteers is 1.95 m^2^ while the BSA of Chinese male volunteers is 1.79 m^2^ (Barter et al, 2013). A larger BSA corresponds to a greater blood volume, which can result in differences in blood concentration following the same drug dose in different ethnic groups. Granisetron is primarily metabolized by CYP3A4 and CYP1A1 enzymes, among which CYP3A4 enzymes have ethnic differences in clinical aspects. The gene frequency of CYP3A slow metabolizers in Chinese is 83%, while the frequency of CYP3A slow metabolizers in Caucasians is 58% (Barter et al, 2013). However, to date no comparative studies have been conducted on the PK of GTDS between the Caucasian and the Chinese populations.

The population pharmacokinetics (Pop PK) approach allows for combining blood concentration data from different clinical groups (Rajman, 2008; Tuntland et al, 2014). The objective of this study is to figure out whether dosage adjustment is necessary for the use of GTDS in the Chinese population. In order to evaluate the pharmacokinetic characteristics of Caucasians and Chinese as much as possible, clinical data from 5 trials (4 Caucasians, 1 Chinese) were included. But the different administration durations and PK sampling points from five trials made it impossible to compare PK parameters by NCA analysis. To address this problem, a Pop PK modeling approach was adopted in this study. The PK data from four experiments on Caucasians were collected and modeled. A comparison of the PK data was conducted to assess the differences in PK between Caucasians and Chinese individuals when using GTDS.

## 2 Materials and methods

### 2.1 Study design

The current study included four Caucasian healthy volunteer trial groups that were modeled using data from the GTDS only. The source of Caucasian healthy subjects’ data was shown in Table 1.

(1) 392MD11/C: A cross-over, open-label, comparative pharmacokinetic study evaluating three different forms of GTDS. A total of 12 healthy white subjects were completed, using the data of 12 individuals given GTDS 34.3 mg/52 cm^2^.
(2) 392D/26/C: A double-blind, placebo-controlled study evaluating the cumulative skin irritation and allergic potential of GTDS. A total of 200 Caucasian healthy subjects were completed, of which 24 subgroups collected PK data before and after the first patch (34.3 mg/52 cm^2^) administration. Only data from this subgroup were used.
(3) 392MD/40/C: A study on the effect of age and BMI on the pharmacokinetics of GTDS. The patch is 34.3 mg/52 cm^2^. A total of 29 Caucasian healthy subjects participated in age-related assessments, and 30 healthy subjects participated in body weight assessments. Data from all 59 subjects were used.
(4) 392MD/43/C: A phase I study aimed at evaluating the effect of exogenous heat sources on the pharmacokinetics of GTDS in Caucasian healthy subjects. A total of 16 healthy Caucasian subjects received treatment A (Sancuso^®^ patch alone) and treatment B (Sancuso^®^ patch + Cura-Heat^®^) in a random order. The patch contained 34.3 mg/52 cm^2^. Only data after treatment A were used.

**TABLE 1:** Participants, dosing regimens and PK sampling plans for studies included in the population pharmacokinetics analysis.

Study code	Subjects	Study design	Dosing regimen	No. Of subjects	PK sampling time	No. Of PK samples
392MD/11/C	Healthy Volunteers (Caucasian)	R/SD/OL	34.3 mg (6 days)	12	Pre-dose and post-dose 6, 12, 24, 48, 72, 96, 120 h	96
392MD/26/C	Healthy Volunteers (Caucasian)	DB/PC/SD	34.3 mg (7 days)	24	Pre-dose and post-dose 8, 24, 48, 72, 96, 120, 144, 168 h	216
392MD/40/C	Healthy Volunteers (Caucasian)	SD	34.3 mg (9 days)	59	Pre-dose and post-dose 8, 24, 48, 72, 96, 120, 144, 168, 192, 216 h	660
392MD/43/C	Healthy Volunteers (Caucasian)	R/SD	34.3 mg (8 days)	16	Pre-dose and post-dose 1, 2, 3, 4, 6, 8, 24, 25, 26, 27, 28, 30, 48, 49, 50, 51, 52, 54, 72, 96, 120, 144, 168, 192 h	400
SP-0102	Healthy Volunteers (Chinese)	R/SD/OL	34.3 mg (6 days)	24	Pre-dose and post-dose 6, 12, 24, 48, 72, 96, 120, 144, 150, 168, 192, 216, 240 h	360

R, randomized; DB, double blind; PC, placebo control; OL, open label; SD, single dose.

### 2.2 Population pharmacokinetic model building

The Pop PK model was built using Phoenix NLME version 1.30 (Cetara, United States), and all parameters were estimated using first-order conditional estimation and extended least squares (FOCE ELS).

First, the semi-logarithmic drug-time curves of GTDS blood concentration data were drawn for exploratory data analysis. One-compartment disposition models were evaluated with first-order elimination. This model was then used to evaluate the most appropriate absorption model. Zero-first order absorption, saturable Michaelis-Menten absorption, and first-order absorption models were investigated. Model discrimination was based on the objective function value (OFV) proportional to −2 times the log likelihood of data. The best model was selected based on OFV, Akaike Information Criteria (AIC), minimization success, and visual inspection of goodness-of-fit plots. Inter-individual variability (IIV) was added exponentially to all parameters according to Eq. (1).

$$\theta_{i}=\theta_{TV}\times e^{\eta i}$$ (1)

where θ_i_ is the individual parameter estimated for the i*th* individual, θ_TV_ is the population value of the investigated parameter, and the parameter η_i_ is the individual deviation from the population parameter value for the i*th* individual. The η is drawn from a normal distribution with mean zero and a variance ω^2^. An additive model, proportional model, and mixed model were included successively to describe the residual variability.

Initial values of the parameters were estimated for Pop PK model using the Naive-pooled method. For covariate analysis, a stepwise forward inclusion (a decrease in OFV of >3.84, *p* < 0.05) and a stricter backward exclusion procedure (an increase in OFV of >6.64, *p* < 0.01) were used as the filtering method. Besides, the biological plausibility and a reduction of IIV were also considered as the main criteria for covariate analysis. All covariates, including age, weight, height, body mass index (BMI), and gender were screened in the model respectively. The effect of continuous covariates was expressed using a power function equation after normalized by the median value of one covariate as Eq. 2. The effect of categorical covariates was represented by exponential equations as Eq. 3.

$$\theta_{i}=\theta_{TV}\times {\left(\frac{{cov}_{i}}{{cov}_{median}}\right)}^{\theta x}$$ (2)

$$\theta_{i}=\theta_{TV}\times e^{\theta xcov}=k$$ (3)

Where covi and covmedian represent covariate values for the ith individual and population median. The k is a categorical variable, and θx is a coefficient used to represent the size of the covariate effect.

### 2.3 Model evaluation and simulation

#### 2.3.1 Model evaluation

The accuracy and stability of the final model were evaluated using the bootstrap method (Bootstrap) with repeated sampling and the visual prediction check method (VPC). The Bootstrap method performs repeated random sampling of subjects in the database 1,000 times, replacing the original dataset with another dataset of the same size but with a different combination of subjects. The median values and 95% prediction intervals gained from Bootstrap were compared to the parameter obtained from the original data set.

The VPC in Phoenix NLME is a Monte Carlo simulation that simulates the plasma concentrations of subjects at specified sampling time points according to fixed values of estimated parameters, IIV, and covariates (Post et al, 2008). The number of subjects per group was the same as the original data. A total of 1000 groups were simulated. From the total simulated data set, the median and the 5th and 95th percentiles of the concentration curves were calculated.

#### 2.3.2 Model simulation

Individual empirical Bayes estimated PK parameters from the final model were used to predict the exposure for the Caucasian population. One thousand replicate trials were simulated according to the design of the GTDS study in Chinese subjects (the same number of subjects in each repeated trial, the same dose drug regimen, and the same sampling time point). The 5th to 95th percentile prediction intervals of the plasma concentration-time curve were calculated with simulated data. The observed concentration points for Chinese healthy subjects were then compared with the 5th to 95th percentile prediction intervals of plasma concentrations for Caucasian healthy subjects in the same graph. The 5th to 95th percentile prediction intervals of 1,000 median plasma concentration-time profiles in Caucasian healthy subjects were compared with the median observed blood concentration curves in Chinese healthy subjects.

If approximately 90% of the observed Chinese plasma concentrations fell within the 5th to 95th percentile prediction interval for Caucasian concentrations and if the median observed curve for Chinese was approximately within the 5th to 95th percentile prediction interval for the simulated median curve for Caucasian, it was considered no significant difference in PK between Chinese and Caucasian populations.

## 3 Results

### 3.1 Population characteristics

Demographic data were summarized in Table 2. A total of 1372 blood concentration data from 112 Caucasian healthy subjects from 4 experimental groups were used for modeling. All included subjects were treated with a single dose of 34.3 mg/52 cm2 GTDS. The demographic characteristics of Caucasian healthy subjects for clinical trials were as follows. The average age was 43.01 ± 17.80 (years). The average weight was 70.54 ± 16.13 (kg). The average height was 169.51 ± 9.69 (cm). The average BMI was 24.39 ± 4.43 (kg/m^2^). The proportion of women was 42.86%.

**TABLE 2:** Demographic and baseline characteristics (mean ± SD).

Study name	392MD/43/C	392MD/40c	392MD/26/C	392MD/11/C	SP-0102
Population	Caucasian	Caucasian	Caucasian	Caucasian	Chinese
n	16	60	24	12	24
Age (years)	31.06 ± 7.86	49.53 ± 20.6	37.63 ± 10.2	37.08 ± 4.62	27.13 ± 4.07
Weight (kg)	64.41 ± 9.73	71.83 ± 19.0	68.27 ± 12.0	76.84 ± 8.67	65.07 ± 5.67
BMI (kg/m^2^)	23.14 ± 2.24	25.19 ± 5.43	23.32 ± 2.58	24.24 ± 2.69	22.56 ± 1.69
Height (cm)	166.50 ± 7.4	168.15 ± 9.5	170.63 ± 10	178.08 ± 3.6	169.88 ± 6.16
Sex-female, n (%)	50.00%	46.67%	50.00%	0.00%	0

A total of 24 Chinese healthy subjects came from one trial. The average age was 27.13 ± 4.07 (years). The average weight was 65.07 ± 5.67 (kg). The average height was 169.88 ± 6.16 (cm). The average BMI was 22.56 ± 1.69 (kg/m^2^). The proportion of women was 0%.

### 3.2 Population pharmacokinetic model building

Firstly, we compared several possible absorption models of GTDS under the assumption of first-order elimination and one-compartment models. The first-order absorption model was found to fit the data best, and thus the first-order absorption model with no lag time was chosen. Subsequently, we compared the AIC (Akaike information criterion) and BIC (Bayesian information criterion) of the one-compartment and two-compartment open models under the first-order absorption and first-order elimination. The AIC and BIC values were found to be lower for the one-compartment model compared to the two-compartment model (one-compartment model AIC: 4895.1498, BIC: 4931.6616; two-compartment model AIC: 4963.0765, BIC: 5020.4522). The clearance (CL2) and volume (V2) of the peripheral compartment in the two-compartment model were several orders smaller in magnitude than the clearance (CL) and volume (V) of the central compartment. In addition, the CV% of all parameters in the one-compartment model was below 15%. A strong correlation was observed between the observations versus population predictions (PRED), as well as between observations versus individual predictions (IPRED). Therefore, a one-compartment model of first-order absorption and first-order elimination was chosen. The exponential model was chosen as the model to describe the IIV and the additive residual model was chosen to describe the unexplained residual variability. In this study, five covariates were analyzed including age, weight, height, BMI, and sex. Following forward and reverse covariate screening, it was shown that these five covariates did not significantly impact the PK parameters.

**FIGURE 1:**
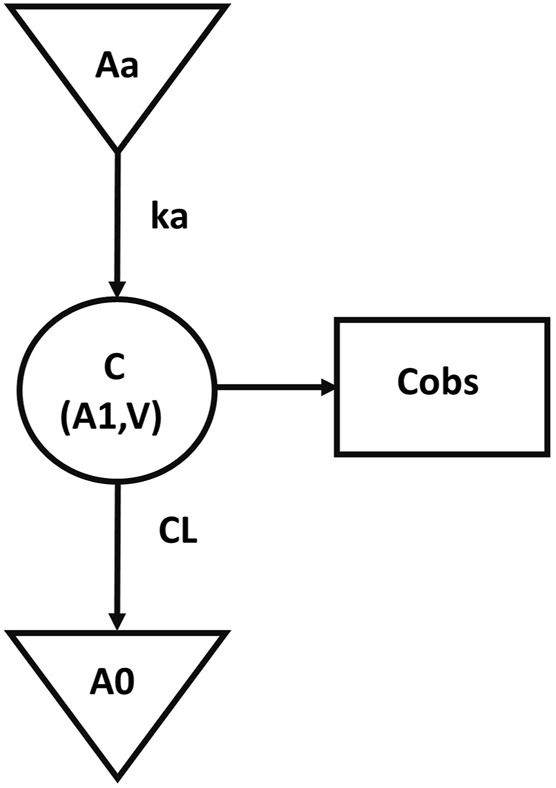
The schematic diagram of final pharmacokinetic model. (Aa, drug dose at absorption site; Ka, absorption constant; C, central compartment concentration; V, central compartment volume; A1, central compartment drug volume; CL, central compartment clearance; A0, cleared drug volume; Cobs, blood The measured value of drug concentration).

The final population pharmacokinetic model for GTDS in Caucasians was a one-compartment model with first-order absorption (no lag time) and first-order elimination without covariates. IIV was described by an exponential model, while residual variability was described by an additive error model. The final pharmacokinetic model schematic diagram (Figure 1) and differential equation (Eqs 4–10) were as follows.

$$\frac{dA_{a}}{dt}=-k_{a}\times A_{a}$$ (4)

$$\frac{dA_{1}}{dt}=-CL{\times C+k}_{a}\times A_{a}$$ (5)

$$C=\frac{A_{1}}{V}$$ (6)

$$C_{obs}=C+\varepsilon$$ (7)

$$k_{a}=tvk_{a}\times e^{\eta ka}$$ (8)

$$V=tvV\times e^{\eta v}$$ (9)

$$CL=tvCL\times e^{\eta CL}$$ (10)

The symbols used in the above formulas are the same as those in the structure model in Figure 1. Eq. 4 described the first-order absorption process, and Eqs 5–6 described the rate of the amount of dose changing in the central compartment. Eq. 7 described the selected additive error model and Eqs 8–10 illustrated the lognormal distribution of the IIV of the parameters Ka, V, and CL. The estimated values and coefficients of variation of the parameters are listed in Table 3.

**TABLE 3:** Parameter estimates of the final population pharmacokinetic model.

Parameter	Estimate (RSE%)	Bootstrap	
		Mean	95% CI
Fixed effect			
Ka (1/h)	0.0179,879 (3.6330732)	0.017966084	0.016469625-0.019216464
V (mL)	6299030 (13.121,334)	6351196.5	4962557.7-8105,176.7
CL (mL/h)	31316.3 (5.5226182)	31042.673	26407.14-36914.471
Random effect			
Ka (1/h)	4.74078E-11 (149.10549)	4.74E-11	0.00000 - 0.00000
V (mL)	1.5462161 (12.10852)	1.5462161	1.179,257-1.9131749
CL (mL/h)	0.25423392 (20.19274)	0.25423392	0.1536138-0.3548540
Residual error			
σ	1.18094 (10.165,277)	1.1706386	0.94536484-1.4335495

### 3.3 Model evaluation

**FIGURE 2:**
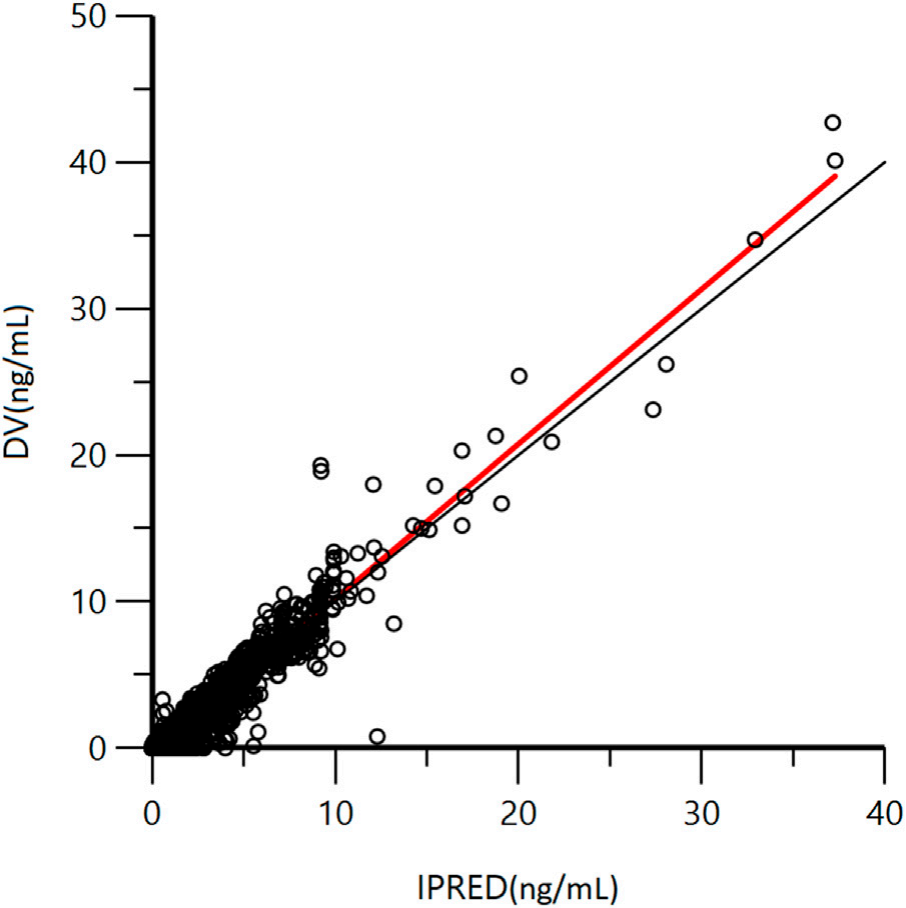
Goodness-of-fit plot of individual predicted concentration (IPRED) and measured concentration (DV) of GTDS.

**FIGURE 3:**
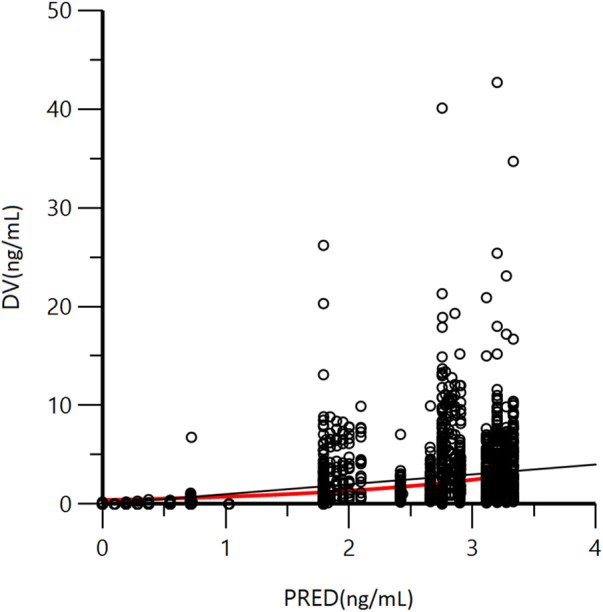
Goodness-of-fit plot of population predicted concentration (PRED) and measured concentration (DV) of GTDS.

**FIGURE 4:**
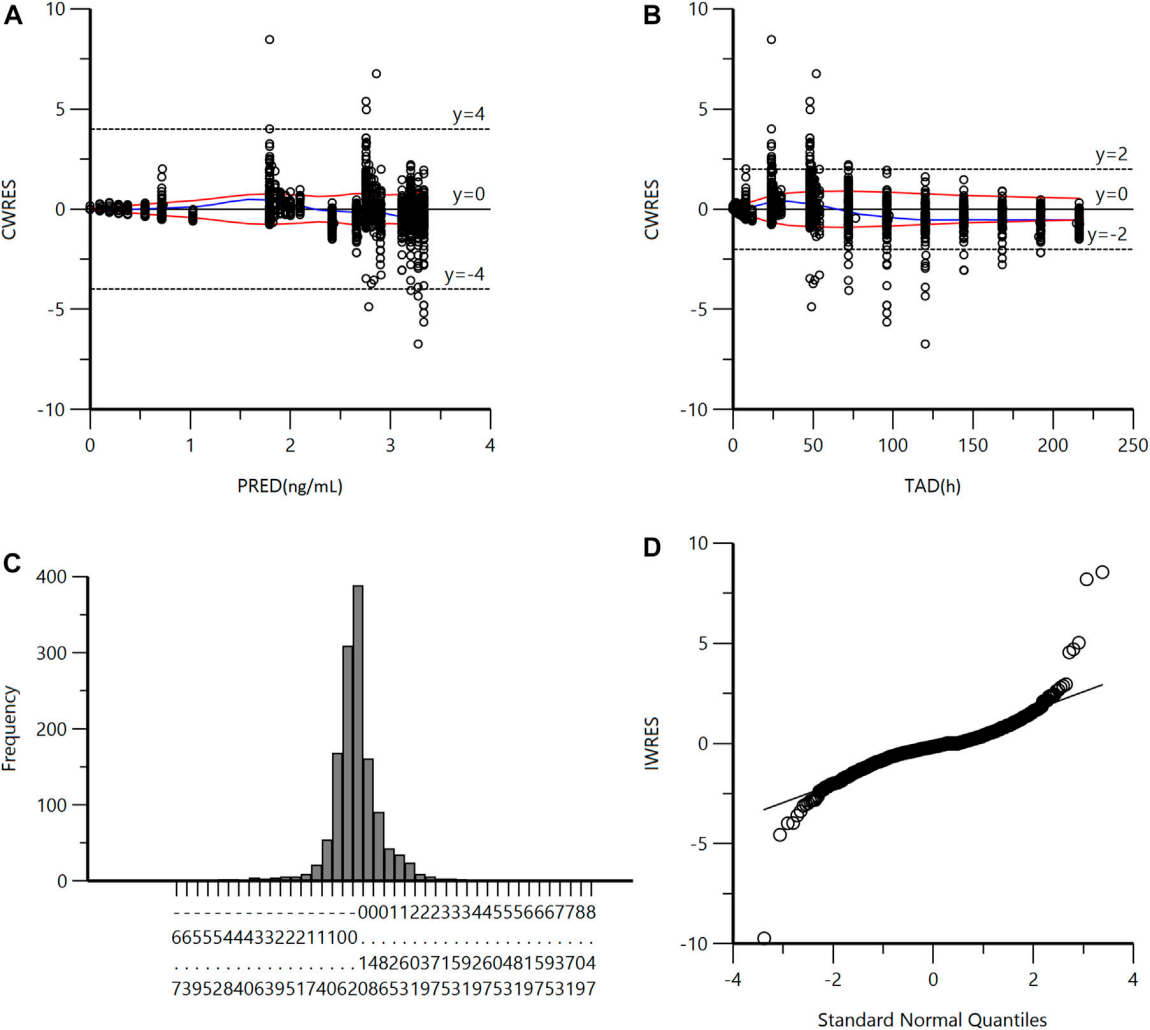
**(A)** Conditional Weighted Residuals (CWRES) vs Population Predicted Concentration Plot (PRED). **(B)** CWRES vs Time (TAD). **(C)** CWRES Distribution. **(D)** CWRES vs Standard Normal Quantiles.

A goodness-of-fit plot for the final model was shown in Figure 2. Both individual and population predictions closely aligned with the observations, as shown in Figure 2 and Figure 3. The trend of the scattered points in Figure 2 was close to the unit line (y = *x*). The relationship between the conditional weighted residual (CWRES) value versus PRED, as well as the relationship between the CWRES value versus time, demonstrated a symmetrical distribution of points near the line passing through zero, most of the points were within −4 and +4 units, as shown in Figure 4A,B. The distribution fitted by CWRES was almost symmetrical and the CWRES fitted standard normal quantiles well, as shown in Figure 4C and Figure D. No evidence of offset was observed.

**FIGURE 5:**
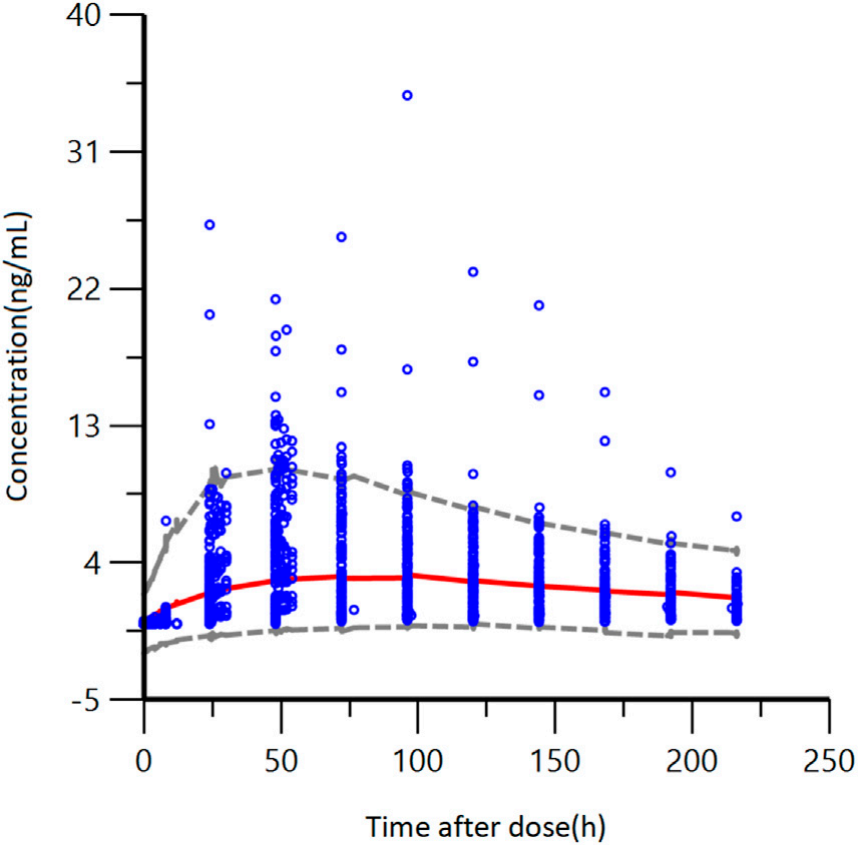
VPC results of the final pharmacokinetic model, blue points represent measured data. The gray dashed lines represent the 5th and 95th percentiles; the red line corresponds to the 50th percentile.

The accuracy and stability of the final model were evaluated using Bootstrap and VPC analyses. The results of the VPC test were presented in Figure 5. These figures showed that most of the observed plasma concentration data fell within the 5th to 95th percentile range of the simulated data. For the results of VPC, blue dots represented measured data. The top and lower lines corresponded to the 5th and 95th percentiles of observed data; the middle line corresponded to the 50th percentiles of the observed data. The results of the Bootstrap method are listed in Table 3 **.** The PK parameters from the final Pop PK model were all included in the 95% confidence interval of the bootstrap procedure.

In conclusion, these figures showed that the final model developed for GTDS well described the observed data from Caucasian healthy subjects. The final model of GTDS encompassed a one-compartment, open model with first-order absorption (no absorption delay) and first-order elimination. No significant covariates were identified, and between-individual variability was described by an exponential model, with residuals described by an additive error model.

### 3.4 Model simulation

**FIGURE 6:**
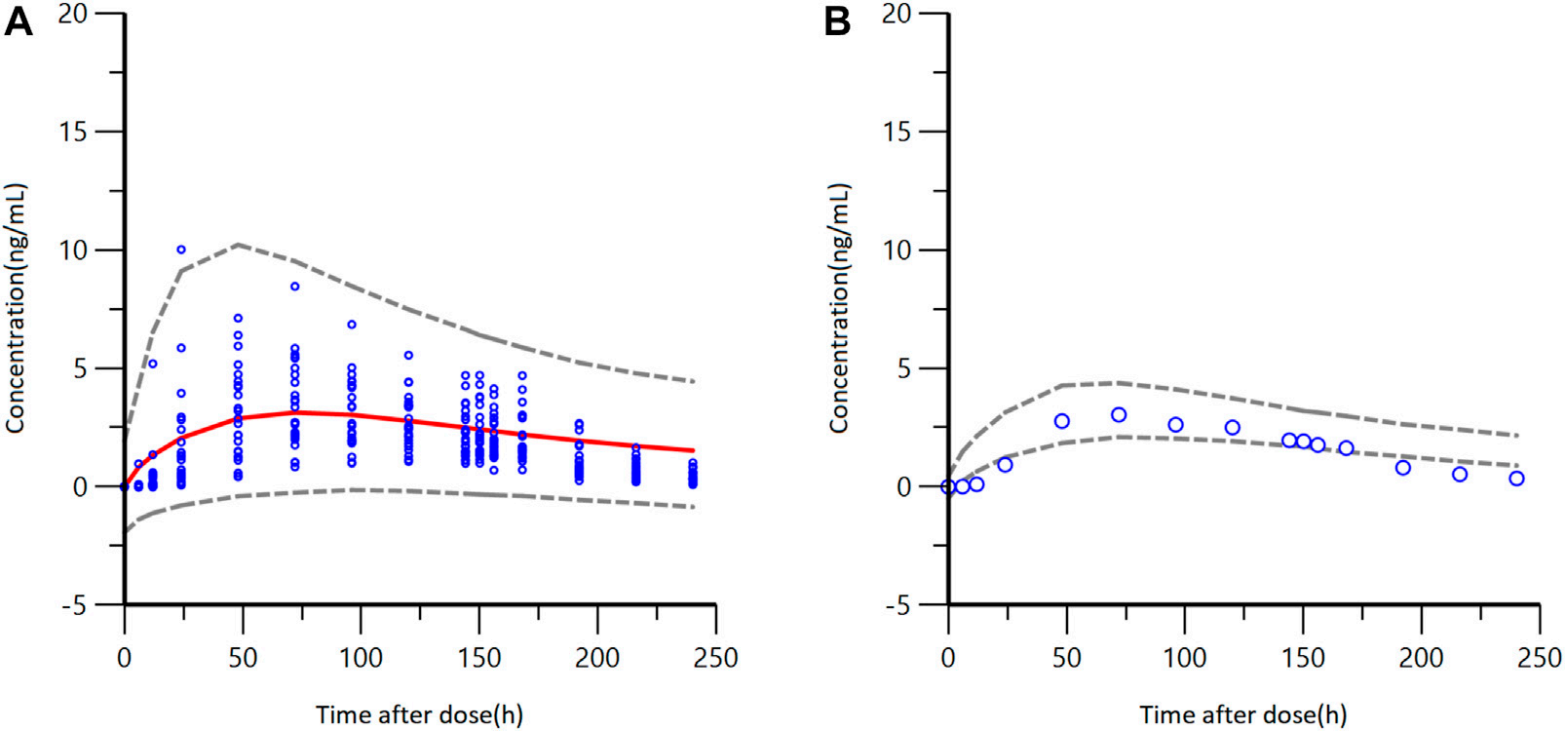
GTDS plasma concentration-time curves between observed Chinese healthy subjects and a simulated Caucasian healthy control group (*N* = 24*1000) after a single administration of GTDS 34.3 mg/52 cm^2^ for 6 days **(A)** Blue circles are observed Chinese concentrations. Red solid line represents the median of Caucasian predicted concentrations, and the gray dashed lines represent the 5th and 95th percentiles of simulated Caucasian data (*N* = 24*1000). **(B)** Blue circles are observed Chinese median concentrations. Gray dashed lines represent the 5th and 95th percentiles from the simulated median concentration data of Caucasian.

The final model was utilized to simulate the pharmacokinetics parameters in Caucasian healthy subjects. The difference in PK parameters between Caucasian and Chinese healthy subjects was studied. After a single administration of GTDS 34.3 mg/52 cm^2^ over 6 days, the comparison of GTDS plasma concentration-time curves between Chinese healthy subjects and simulated Caucasian healthy control group (*N* = 24*1000) is shown in Figure 6 **.** Most of the plasma concentrations measured in Chinese subjects fell within the 5th to 95th percentile prediction interval of predicted Caucasian subjects’ data. Furthermore, the median plasma concentration-time curves of GTDS plasma concentrations measured in Chinese subjects were close and partially overlapped with the 5th to 95th percentile prediction intervals of the Caucasian median plasma concentration-time curve from simulated 1000 Caucasian controls (*N* = 24 each) **(** Figure 6 **)**.

Predicted PK parameters of Caucasians versus observer Chinese PK parameters were listed in Table 4. The result showed that the median values of AUC and C_avg_ in Chinese healthy subjects were contained within the 5th to 95th percentile prediction intervals of 1000 Caucasians median value obtained from simulated data. The median value of t_max_ for Chinese healthy subjects also fell within the 5th to 95th percentile prediction interval for the Caucasian subjects, which was located at the lower limit. The median C_max_ of Chinese healthy subjects was slightly below the 5th to 95th percentile prediction interval of median C_max_ for Caucasians.

**TABLE 4:** Predicted versus observed PK parameters in Chinese and Caucasian population.

PK parameters (units)	Single dose of transdermal granisetron 34.3 mg/52 cm^2^	
	Chinese	Caucasian
AUC_last_ (hr*ng/mL)	474.07	569.94 [431.18,728.17]
C_avg(_ng/mL)	2.67	2.79 [1.98,3.79]
C_max_ (ng/mL)	3.62	4.71 [3.86,5.73]
T_max_ (hr)	72	108 [72,144]

Chinese: median value; Caucasian: median value and (P5:P95).

In conclusion, these findings indicated that there were no significant differences between Chinese and Caucasians in terms of AUC_last_ and C_avg_, which are the two mains primary characterizing *in vivo* exposure, following administration of GTDS.

## 4 Discussion

To bridge the GTDS data in Caucasian and Chinese populations, the comparison based on the modeling method can effectively integrate the data from previous foreign studies and established a population pharmacokinetic model in Caucasians. This model simulated a large sample of Caucasian subjects as a control group according to the Chinese study design. The method in this study overcame the limitations of descriptive statistical methods in several ways. First of all, the developed population pharmacokinetic model can integrate the data of previous studies with different drug administration and sampling designs. The modeling method expanded the sample size and optimized the sampling error that the small sample studies might take (Rajman, 2008). Besides, more data gathered makes for a better understanding of the IIV of each PK parameter. Secondly, simulating a large-sample size Caucasian control group enabled a visual representation of the PK distribution in the Caucasian population. Last but not least, the modeling method took into account the demographic differences between the two ethnically sampled populations. The influence of potential covariates such as demographic distribution on PK parameters was explored. This led to more reliable scientific conclusions about racial differences in PK with greater confidence.

In the present study, a Pop PK model was developed based on data from Caucasian healthy volunteers. The final model for the GTDS was a one-compartment, open model with first-order absorption (no lag time) and first-order elimination, without covariates. The population pharmacokinetic model was applied to simulate the large-sample Caucasian control group with the Chinese drug regimen, and compared with the measured data of Chinese healthy people. The results showed that the PK parameters of GTDS were similar between Chinese healthy subjects and Caucasian healthy subjects. Combined with the therapeutic window of granisetron, it is considered that the use of GTDS in the Chinese population may not require dose adjustment.

This study is the first study to report the difference in the pharmacokinetics of GTDS between Chinese and Caucasians. According to the study results, the median C_max_ of Chinese healthy subjects was slightly lower than the 5th to 95th percentile prediction interval of the median C_max_ in Caucasians. The difference may arise mainly from the process of absorption that occurred in human skin. Eston reached a study comparing the subcutaneous adipose tissue thickness in Chinese and British men (Eston et al, 1994). It mentioned that the subcutaneous adipose tissue thickness of biceps and triceps in British men was greater than that of Chinese men, as measured using both Caliper and Ultrasonography (US) methods. The mean values of fat from biceps and triceps in British people were 4.2 mm and 5.6 mm, respectively, as determined by US. The mean values of fat from biceps and triceps in Chinese people were 3.9 mm and 4.2 mm by US. When it comes to absorption of granisetron, which has a log P of 2.8 (Inoue and Yuasa, 2014), the greater the thickness of subcutaneous adipose tissue, the more granisetron can permeate through skin. Thus, the higher subcutaneous adipose tissue thickness in Caucasians might be one of the reasons for the higher C_max_ observed in our study. Besides, Asians have been found to have the lowest baseline of trans-epidermal water loss (TEWL) compared to Black, Caucasian, and Hispanic people (Wesley and Maibach, 2003). Dryer skin is associated with a risk of reduced permeability. This may further explain the lower C_max_ for GTDS in Chinese individuals in our study. However, due to the lack of skin TEWL data of Asians, especially Chinese individuals, there is no consensus on which population gained the least TEWL level.

The properties of the skin not only influence the absorption of GTDS but also have implications for drug safety. Stratum corneum barrier function analysis among Caucasian, Black and Asian subjects has shown that Asian people have more sensitive stratum corneum when exposed to exogenous chemicals (Kompaore and Tsuruta, 1993). The sensitivity may also reach a difference when absorbing the GTDS. However, due to ethical considerations and feasibility, fundamental absorption data from humans is difficult to obtain. The current physiological-based pharmacokinetic modeling method can be used as a substitute to predict the systemic exposure process after skin administration (Chetty et al, 2018). This method is being developed and previously used in Diclofenac. Diclofenac was absorbed transdermally by applying it to the forearm of volunteers. A mechanistic dermal absorption model was built with stratum corneum, viable epidermis-dermis, and the blood compartment along with human dermal physiological parameters such as race and gender. The model was well validated with clinical pharmacokinetics data and simulated the key parameters of transdermal absorption, such as the stratum corneum-water partition coefficient. Besides, it predicted the Diclofenac blood concentration which is close to the clinical observed values (Polak et al, 2012). Given the slight difference in the C_max_ of GTDS between Chinese and Caucasians in this study, it would be feasible to incorporate specific skin-related parameters of Chinese subjects and Caucasians into PBPK modeling to conduct research and interpretation. It is important to note that overall, the difference in C_max_ and t_max_ fell within an acceptable range.

This study took into account the demographic differences between the two racial sample populations. To explore the influence of potential covariates, such as demographic distribution on PK parameters, the covariates screened included age, weight, height, BMI, and sex. The results of covariate analysis are consistently aligned with previous research (Howell et al, 2009). In addition, other research articles pointed out that renal function (Carmichael et al, 1989; Howell et al, 2009), short-term exogenous heat (such as exposure to hot water, Sun, etc.), and skinfold thickness (Gilmore, 2013) were not covariates of GTDS PK parameters.

None of the aforementioned covariates were found to significantly impact the PK parameters, the factor of gender still needs to be noted. In a study comparing the efficacy of GTDS and oral preparations, 313 subjects were randomly divided into two groups, patch and oral treatment groups. The primary outcome measures were the rate of complete control (CC) of nausea and vomiting from chemotherapy initiation until 24 h after final administration. In the female subgroup of the study, when randomized to receive oral or patch treatment, the CC of the transdermal patch was significantly higher than that for oral granisetron (*p*=0.0268) (Yang et al, 2016). Since plasma concentrations were not measured in this study, it was hard to know whether higher effectiveness in the subgroup of women is due to higher plasma concentrations. In Duggan’s study, it was mentioned that women had higher plasma concentrations of granisetron than men when given GTDS, although not statistically different (Duggan and Curran, 2009). In the search for covariates, it is suggested that more attention be paid to including data related to cancer patients’ physiological characteristics and multidrug regimens for exploration. In a study comparing the efficacy of GTDS with oral preparations in patients, it was also noted that in the subgroup of the cisplatin-contained regimen, the CC of the patch group was significantly higher than that of oral granisetron. Since GTDS is mainly used for the prevention and treatment of CINV in cancer chemotherapy patients and a cisplatin-contained regimen is the main therapeutic drug for chemotherapy, the advantages of transdermal patches over oral preparations are obvious. Currently, the relationship between the PK of granisetron and the efficacy of the drug is not fully understood, but it has been reported that granisetron can prolong the QT interval in a dose-dependent manner (Mason et al, 2012). Hence, it is meaningful for GTDS to search possible covariates continuously, not only to improve the dosing regimen but also to prevent potential safety issues.

Granisetron is mainly metabolized by CYP3A4 and CYP1A1 enzymes, and CYP3A4 enzymes show ethnic differences in clinical aspects. The gene frequency of CYP3A slow metabolizers in Chinese is 83%, while in Caucasians, it is 58% (Barter et al, 2013). It is suggested that although the healthy population did not show racial differences in this study, the dosage adjustment among patients of different races needs more blood concentration data for further exploration.

Admittedly, lacking the PK data on GTDS in Chinese healthy females, the small sample size of the Chinese population, and the generally young age of the Chinese population were the main limitations of this study. When the comparison was made between Caucasian healthy male and female subjects and Chinese healthy male subjects in our study directly, the gender proportional differences between these two populations were carefully considered. The gender difference was designated as one of the covariates when analyzing the Caucasian data. In the large sample of healthy Caucasian individuals presented in the manuscript of our study (*n*=111), covariate screening was performed, but gender was not included in the final covariate results. In Howell’s study, which included a larger sample size of Caucasian healthy subjects (*n* = 48) and cancer patients (*n* = 793), reported the same finding that gender didn't affect GTDS PK (Howell et al, 2009). This result convincingly indicates that gender doesn't have a significant influence on the pharmacokinetics of GTDS. Besides, during the process of its metabolism, the dose fraction metabolized via CYP3A4 only counts for 0.05% so the expression difference of CYP3A4 hardly affects the PK of GTDS (Dallmann et al, 2018). In the consideration of CYP enzymes influenced by age, it was excluded in the covariate analysis section, the dosage adjustment among patients of different ages needs more blood concentration data for further exploration. It is important to note that our study focused on healthy Chinese and Caucasian populations, and the potential effects of multidrug regimens in patients were not considered. According to the literature, the incidence of drug interactions between drugs and GTDS is generally low (Duggan and Curran, 2009). As some anticancer drugs are metabolized by CYP enzymes, inhibitors, and inducers may impact the elimination of 5-HT3 receptor antagonists. At present, there are no definitive drug-drug interaction clinical studies evaluating the PK interactions of GTDS with other drugs (Gan, 2005). Further drug-drug interaction studies might be necessary for GTDS and multidrug regimens.

Our study explored the potential ethnicity effect of potential subjects using the GTDS and broadened the drug’s applicability to a wider range of countries. Although the pathogenesis of CINV has not yet been fully elucidated, the 5-hydroxytryptamine type 3 (5-HT3) receptor pathway has been proven to be one of the involved pathways (Yang et al, 2016). As compared with other 5-HT3 receptor antagonists such as ondansetron, dolasetron, and tropisetron (Gan, 2005). GTDS, as the only one in the category of 5-HT3 receptor antagonists that has a patch formulation (NCCN., 2023), provides a non-invasive option for patients who have difficulties with oral or intravenous administration. Additionally, GTDS offers the advantage of sustained therapeutic effects, as the patch can be worn on the arm for up to 7 days, maintaining a relatively steady plasma concentration even after reaching the C_max_ for a duration of 96 h (Keating et al, 2012). However, it takes more time for GTDS to reach the effective concentration due to the relatively slow absorption of patches. And therefore, patch administration is required before chemotherapeutic drugs are administered sometimes. Furthermore, in some patients prolonged patch administration may cause local reactions.

## 5 Conclusion

The plasma concentration of GTDS measured by Chinese subjects was mostly within the 5th to 95th percentile interval of the plasma concentration predicted by the established Caucasian Pop PK model. There were no significant differences between Chinese and Caucasians for the two main parameters AUC_last_ and C_avg_ that characterize the exposure in humans after patch administration.

## Data Availability

The original contributions presented in the study are included in the article; further inquiries can be directed to the corresponding author.
